# Gene Expression and Enzyme Kinetics of Polyphenol Oxidases in Strawberry and Their Possible Involvement in Enzymatic Browning Reactions in Strawberry Nectar

**DOI:** 10.3390/foods14122064

**Published:** 2025-06-11

**Authors:** Alberto Zavarise, Ibrahim Rabeeah, Christian Molitor, Mahboubeh Davoudi Pahnekolayi, Viktoria Gruber-Schmidt, Andrea Winter, Klaus Olbricht, Christian Haselmair-Gosch, Karl Stich, Manfred Goessinger, Heidi Halbwirth

**Affiliations:** 1 Research Group for Phytochemistry and Biochemistry of Natural Compounds, Institute of Chemical, Environmental and Bioscience Engineering, Technische Universität Wien, 1060 Wien, Austria; 2 Hansabred GmbH & Co. KG, Radeburger Landstraße 12, 01108 Dresden, Germany; 3 Department of Fruit Processing, Federal College and Institute for Viticulture and Pomology, Wiener Straße 74, 3400 Klosterneuburg, Austria

**Keywords:** polyphenol oxidase, enzyme kinetics, browning, gene expression, *Fragaria vesca*, *Fragaria* × *ananassa*

## Abstract

The browning of fruit juices and nectars is a common issue in the beverage industry and is a particular problem in strawberry nectars, where it significantly reduces the shelf-life. Polyphenol oxidases (PPOs), which are multicopper enzymes responsible for the oxidation of a wide plethora of polyphenols in plants, have been widely assumed to be involved in the enzymatic browning of strawberry nectar. To investigate the possible involvement of PPOs, the substrate specificity of four recombinant PPOs and their gene expression pattern in 10 cultivars of *Fragaria* × *ananassa* at five ripening stages were determined. This allowed us to obtain adequate amounts of enzymes to study them independently and without interfering matrix effects. All four PPOs possess monophenolase activity, which was particularly high for PPO4. PPO3 did not show sufficient stability for the kinetic studies. The other three showed a high preference for the flavan 3-ol catechin with a 2-fold higher catalytic efficiency compared to dopamine for PPO1 and PPO2. At a neutral pH, they also showed activity with cyanidin but not with pelargonidin, which is the prevalent anthocyanidin type in strawberry. The enzymes showed a high affinity but only low turnover rates for the dihydrochalcone phloretin, resulting in an inhibitory effect that was strong enough to extend the shelf-life of the strawberry nectar by one week if phloretin was added in high concentrations (600 µM). PPO1 and PPO2 were prevalently expressed in all fruit stages. The gene expression of the four PPOs did not correlate with the color stability of the nectars of the 10 varieties and also showed a random expression pattern during fruit development. The limited activity in acidic conditions and the low substrate specificity for pelargonidin does not point to a crucial role for PPOs in the browning of strawberry nectar, but the high catalytic efficiency with catechin as a substrate could contribute to anthocyanin degradation via mechanisms such as copolymerization.

## 1. Introduction

Strawberries are popular fruits, sought after for their characteristic vibrant red color, aromatics, and sweet, slightly acidic taste. According to the FAO, the market for whole fruit and processed products has been growing worldwide since the early 2000s with an increase from 9 to 10.4 million tons from 2020 to 2023 [1]. Strawberries are appealing not only through their sensorial properties but also their nutritional value. They are low in caloric content and rich in vitamin C and folate, fibers, and minerals (calcium, iron, magnesium, potassium), but, most importantly, have abundant antioxidants, such as hydroxycinnamic acids, anthocyanins, flavonols, and flavanols [2,3,4]. The abundance of polyphenols, while of great health benefit for humans, might play a negative role in the shelf-life of the product [5,6]. The browning process in strawberry, raspberry, and blueberry juices and nectars is a technological challenge with a severe impact on shelf-life, even though it is relatively slow when compared with other fruits, such as bananas, and typically takes weeks to be noticeable. Interestingly, it has been demonstrated to be cultivar-specific [7,8]. While color additives could seem an easy solution to the problem, due to EU regulations, they are not a feasible alternative. For the production of nectars, only water, sugar, citric acid, and strawberries are allowed [9], resulting in only a few shelf-stable products available on the market [10].

**Figure 1:**
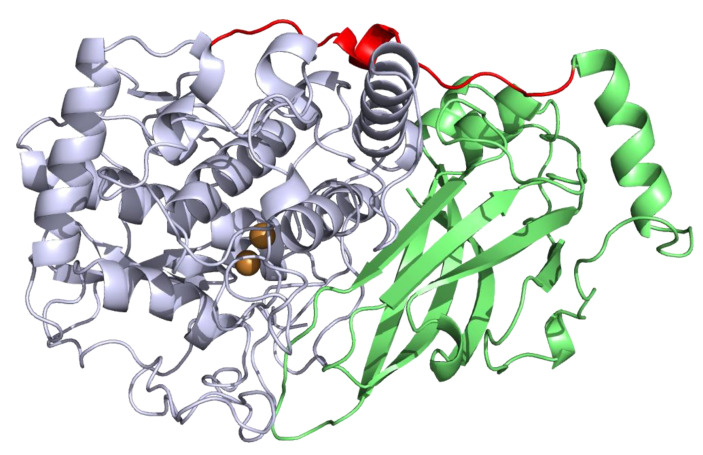
Protein model of polyphenol oxidase (PPO) 2 from *Fragaria vesca* created with Alphafold2 [26]. Active part in faint lilac, C-terminal shielding domain in green, linker region in red, and copper ions in orange.

For the past three decades, a lot of work has been carried out on identifying the cause of this slow browning, which occurs despite pasteurization technology. Enzymatic and non-enzymatic mechanisms have been suggested to contribute to the process [6,11,12]. The successful application of enzymatic inhibitors supports the involvement of enzymes. Polyphenol oxidase (PPO), peroxidase, and β-glycosidase [13,14,15] have been suggested to be responsible for enzymatic browning, with many clues pointing to PPO [9,16,17]. PPO is a multicopper enzyme present in plants, fungi, bacteria, and animals belonging to the oxidoreductase class that oxidizes mono- and polyphenolic substrates [18]. In plants, they fulfil multiple roles contributing not only to defense, but also to the biosynthesis of secondary metabolites and pigments [19,20,21,22]. The structure of the enzyme (Figure 1) shows two conserved tyrosinase domains containing two copper centers (CuA and CuB) that make up the active site and a conserved C-terminal shielding domain that prevents undesired activity [23,24,25].

Most PPOs contain a signal peptide for translocation to the thylakoid lumen, where they are present in a latent or dormant state [27,28,29]. Mechanical grinding, diseases, pathogenic attack, cell senescence, and herbivore feeding causes cell disruption, leading to enzyme activation by proteases, which recognize and cleave a specific peptide sequence present on a linker region that delimits the end of the active part of the enzyme (CuA and CuB) from the shielding domain (C-terminal domain) [23,24,25,27,28,29,30].

**Figure 2:**
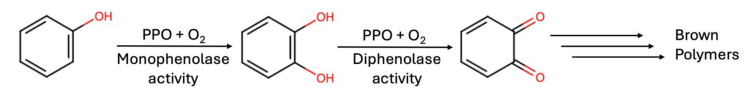
Simplified scheme of the oxidation of mono- and polyphenols catalyzed by polyphenol oxidase (PPO). Monophenolases catalyze both reactions, and diphenolases only the second.

This linker region contains two motifs, DWL and KFDV, that have been shown to be extremely conserved among PPOs [25]. Upon release from the chloroplast, the active enzyme oxidizes polyphenolic substrates to *o*-quinones [31] (Figure 2), which are highly reactive and tend to cross-polymerize with other *o*-quinones and proteins, leading to the formation of brown pigments that help seal the wound and inactivate microbial proteins [32]. In living cells, this reaction does not occur due to the physical separation of the enzyme from the vacuoles and cytoplasm, where pigments and other secondary metabolites are stored [33].

The browning reaction, while vital for the plant, is a technological challenge during food processing and the preparation of products like juices, nectars, and purees. It reduces the nutritional value of the products and taints the characteristic bright red color, thus making them unappealing to consumers and negatively impacting sales and profit [9]. Industry has adapted by trying to mitigate this phenomenon by inactivation of the enzyme via pasteurization and the addition of naturally occurring compounds like citric acid to slow down the oxidation process. The traditional breeding of cultivars with lower PPO activity has also been a successful method to reduce browning, along with the genetic engineering of PPO-deficient fruits, as in the case of Arctic apple [34].

Over the past two decades, significant efforts have been made to reduce enzyme activity using both conventional and non-conventional methods, including pasteurization, high-pressure processing, pulsed electric fields, and ohmic heating. However, little attention has been given to identifying the root cause of the issue. We focused on the gene expression of four PPO isoforms in strawberry with the aim to evaluate their impact on the browning reactions. These four *PPO* genes have been recently identified in wild strawberry (*Fragaria vesca*) [35], yet no study has focused on their kinetic properties or expression patterns in fruit.

In this study, all four *PPO* genes were recombinantly expressed in *E. coli*, purified, and kinetically characterized in vitro using the most abundant flavonoids in strawberries to demonstrate their efficiency with naturally occurring substrates. Additionally, the expression patterns of these genes were analyzed in cultivated strawberry (*Fragaria × ananassa*) using RT-qPCR across five ripening stages in 10 different cultivars/lines to investigate if the *PPO* expression levels correlate to the color stability of the nectars made from different varieties and if a correlation with the observed higher stability in nectars [6,36] made from overripe strawberries can be observed.

## 2. Materials and Methods

### 2.1. Chemicals

All chemicals used for the preparation of buffers and standard solutions were of analytical grade. Substrates for kinetic analysis were obtained from various manufacturers. Catechin (≥99%), catechol (≥99%), chlorogenic acid (95%), dopamine (≥98%), L-DOPA (≥98%), and tyramine hydrochloride (98%) were purchased from Sigma-Aldrich (Merck, Darmstadt, Germany). Phloretin (98%) and isopropyl-β-d-thiogalactopyranoside (IPTG) was purchased from Thermo Fisher Scientific (Waltham, MA, USA). Phloretin (95%), which was used as an additive in nectar preparation, was obtained from Layn Natural Ingredients (Pudong District, Shanghai, China).

### 2.2. PPO Genes and Plasmid Construct

The sequences from *Fragaria vesca PPOs* were obtained from the NCBI database with the gene accession codes as follows: *PPO1* (XM_004293480), *PPO2* (XM_004293478), *PPO3* (XM_004303349), *PPO4* (XM_004293515). Protein sequences were aligned with the sequence of latent PPO from *Malus domestica* on the Clustal Omega Ebi webserver v 1.2.2 (https://www.ebi.ac.uk/jdispatcher/msa/clustalo, accessed on 25 March 2021) to identify the transit peptides. Nucleotide sequences without transit peptides were codon-optimized for *E. coli* with the GeneArt project manager program from ThermoScientific and ordered for cloning into pET28 vector at Twist Bioscience (https://www.twistbioscience.com, accessed on 8 April 2021). Expression of *PPO1*, *PPO2*, and *PPO4* required modification of the plasmid construct due to lack of expression. Briefly, the 6xHisTag was moved to the N-terminus from the C-terminus and non-codon-optimized cDNA sequences for PPO2 and PPO3 were used for successful transcription of the enzyme in the host.

### 2.3. Protein Expression and Purification

Chemically competent *E. coli* BL21(DE3) cells were transformed with the plasmid of interest and a single colony was used to start a 20 mL overnight culture in LB media. The next morning, the entire starter culture was used to inoculate 500 mL of TB media. After shaking (200 rpm) at 37 °C until OD_600_ = 0.6–0.8, expression was induced with 1 mM IPTG, and 1 mM CuSO_4_ was supplemented for metal occupancy of the active site. After 20 h at 18 °C and 200 rpm shaking, cells were harvested by centrifugation (Beckman Coulter, Brea, CA, USA) at 4600× *g* for 10 min a 4 °C. The resulting pellet was transferred to a 50 mL conical tube and flash-frozen in liquid nitrogen; cell pellets were stored at −80 °C until use. Cell lysis was performed on ice with a Sonicator Cell Disruptor Q125 (Qsonica, Newtown, CT, USA). Cells were resuspended in a 1:5 ratio with lysis buffer and the sonicator set to a cycle of 15 s ON and 45 s OFF for a total of 12 min of ON time. Insoluble parts were pelleted at 4800× *g* for 50 min at 4 °C. Supernatant was loaded on a HisTrap FF 5 mL column with an Äkta Purifier 10 FPLC (GE Healtcare, Uppsala, Sweden). Elution fractions were concentrated and buffer exchanged in a Vivaspin concentrator with a 30 kDa cut-off size. Purified protein was stored in 20% glycerol at −80 °C.

### 2.4. SDS-PAGE and Western Blotting

Protein samples for SDS-PAGE were prepared by mixing them with reducing Laemmli Buffer in a 1:4 ratio and by incubating for 10 min at 95 °C. They were then separated on a 12 % acrylamide gel in a Bio-Rad Tetra system (BioRad Laboratories, Hercules, CA, USA). Color Prestained Protein Standard, Broad Range (New England Biolabs, Ipswich, MA, USA) was used as a size standard. Trans-Blot Turbo Transfer System (BioRad Laboratories, Hercules, CA, USA) was used to transfer the samples from the gel to a PVDF membrane (Trans-Blot Turbo^TM^ Transfer Pack, BioRad Laboratories, Hercules, CA, USA). The membrane was blocked with a phosphate-buffered saline (PBS) solution containing 2 % (*w/v*) bovine serum albumin for 1.5 h followed by two 10 min washes in PBS buffer with 0.4 % (*v/v*) Tween-20. AntiHis antibody conjugated to alkaline phosphatase was incubated in 1:5000 dilution for 1.5 h and washed briefly twice with 10 mL of PBS buffer with 0.4 % (*v/v*) Tween-20. Color development was achieved with 5-bromo-4-chloro-3-indolyl-phosphate and nitro blue tetrazolium in alkaline phosphatase buffer.

### 2.5. Enzyme Assays

Enzyme assays were performed at 25 °C on a Shimadzu UV-1800 spectrophotometer (Kyoto, Japan) (for wavelength, refer to Table 1) with a total reaction volume of 1000 µL. All tests were run in technical triplicates. Briefly, 950 µL of buffer containing the SDS and substrate were mixed with 5 µL of the same buffer containing the enzyme in a cuvette with a 1 cm path length. The increase in absorbance was recorded and ΔAbs/60 s was calculated in the linear range of the reaction.

The pH optimum was tested in the presence of 1 mM SDS and 5 mM tyramine, which is a standard substrate for PPOs [37]. The pH values ranged from 3 to 9 with a 0.5 pH step increase. For the range 3 to 5.5, 50 mM citrate buffer was used; pH 6 to 6.5, 50 mM phosphate buffer; and from pH 7 to 9, 50 mM Tris-HCl buffer. Enzyme quantities were 1 µg for PPO1 and PPO2, 3 µg for PPO4, and 30 µg for PPO3.

SDS dependency was tested in 50 mM Tris-HCl buffer (pH 7) in presence of 10 mM catechol with SDS concentration at 0.1 mM, 0.3 mM, 0.7 mM, 1 mM, 2 mM, 3 mM, 5 mM, 10 mM, and 20 mM. Enzyme amounts used were 0.1 µg for PPO1 and PPO2, 0.5 µg for PPO4, and 1 µg for PPO3.

### 2.6. Kinetic Studies

PPO1, PPO2, and PPO4 kinetics were calculated for five naturally occurring compounds (catechin, chlorogenic acid, dopamine, L-DOPA, phloretin) and two synthetic standard substrates for PPO (catechol, tyramine). The substrates were tested at different concentrations in 50 mM Tris-HCl buffer pH 7, with an SDS concentration of 0.7 mM for PPO1 and PPO2 and 1 mM for PPO4. The final volume in the cuvette was 1000 µL with a fixed volume of 5 µL of enzyme solution. Enzyme amounts and respective wavelengths for each substrate used are shown in Table 1. Data analysis for calculation of V_max_, *K_M_*, and V_max_/*K_M_* was carried out on OriginPro software (Version 2023, OriginLab Corporation, Northampton, MA, USA).

Additionally, pelargonidin and cyanidin at 0.5 mM concentrations were tested for activity at both pH 7 (optimal pH) and pH 3.5 (pH of standard nectars) [14,38]. For this assay, the oxygen consumption was measured directly in the cuvette with a FireSting-O_2_ sensor from PyroScience (Aachen, Germany) due to noise at 540 nm derived from substrate instability in the buffer [39]. The assay was carried out by briefly mixing 950 µL of buffer containing substrate and detergent with 5 µL of enzyme solution and immerging the instrument sensor in the cuvette right away. No kinetics were determined for PPO3 due to its instability.

### 2.7. Plant Material

Fruits from breeding lines 90677, 90886, 190826, 190828, 210395, cv. Diamante, cv. Dreistetten, cv. Elsanta, cv. Macheraus Marieva, and cv. Zuckererdbeere Sotschi were harvested at Hansabred GmbH & Co. KG (Dresden, Germany) in June 2024. The selection of these cultivars was based on previously obtained data (Goessinger, unpublished) and the availability of these cultivars. As there are no truly slow-browning cultivars that are also economically viable presently available, clones of four promising lines were selected for their slow browning properties (90886, 190826, 190828, and 210395), along with four more traditional cultivars with medium stability (cv. Diamante, cv. Dreistetten, cv. Macheraus Marieva, and cv. Zuckererdbeere Sotschi), and rapidly browning cv. Elsanta and line 90677 for comparative purposes.

**Figure 3:**
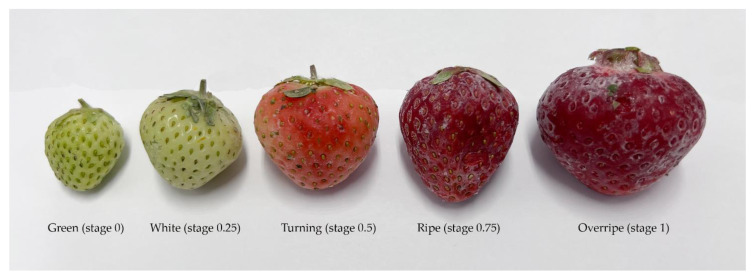
Developmental stages of ripening strawberries investigated in this study. From left to right: green (stage 0), white (stage 0.25), turning (stage 0.5), ripe (stage 0.75), and overripe (stage 1).

Five developmental stages of the fruits (Table 2, Figure 3) were collected: green (0), white (0.25), turning (0.5), ripe (0.75), and overripe (1). Samples were frozen in liquid nitrogen immediately after harvesting and stored at −80 °C.

### 2.8. Nectar Preparation

Fifty kilograms of strawberries (mixed cultivars) were milled using a roller crusher (Wottle, Poysdorf, Austria), then sieved to 1 mm with a sieving machine (Wiesböck, Wien, Austria), and further processed with a colloid mill (Fryma, Rheinfelden, Switzerland). The resulting puree was mixed with a calculated amount of water, sugar, and acid to achieve a pH of 3.5 and 15° Brix. The nectar mixture was stirred and homogenized using a colloid mill, then degassed in a vacuum tank at −0.6 bar for 15 min. Finally, the nectar was filled into 0.2 L glass bottles using a vacuum filler (Rapf & Co., Maria Enzersdorf, Austria). After sealing, the bottles were pasteurized at 85 °C for 20 min.

For each variation, 25 bottles (200 mL each) of nectar were prepared. A control nectar and four variations were tested. The first two variations contained phloretin at concentrations of 30 µmol/L and 600 µmol/L, respectively, added before sealing. In the third variation, the water in the nectar recipe was replaced with commercial clear apple juice. The fourth variation included aubergine powder at a 1:5 (*w*:*v*) ratio, prepared from fresh, peeled aubergine ground into a fine powder using liquid nitrogen. The aubergine powder was incorporated into the nectar before homogenization. All samples were stored in a dark room at 20 °C until further testing.

### 2.9. Color Measurement and Acceptance Factor Calculation

Two bottles of samples were used for each variation to measure color using the CIELAB color system components L* (lightness), a* (red-green), and b* (yellow-blue), which were then used to calculate C* (chroma) and h◦ (hue angle). A Minolta CM-5 spectrophotometer was employed for spectrophotometric measurements (D65, 30 mm, 10°, reflection measurement, gloss excluded; Minolta, Osaka, Japan). Measurement was performed in duplicate for each bottle. The Acceptance Factor (AF) was calculated as previously reported [9], using the following equation: AF = a*/h°. AF values above 0.7 are considered excellent and between 0.7 and 0.5 are considered good, and anything below 0.5 is deemed unacceptable.

### 2.10. RT-qPCR Analysis

Whole fruit samples comprising flesh and achenes minus calyx were ground with liquid nitrogen in an IKA Basic blender and the resulting fine powder was used for mRNA extraction with a µMACS mRNA kit (Miltenyi Biotec, Bergisch Gladbach, Germany). cDNA synthesis was performed with RevertAid^TM^ Reverse Transcriptase (Thermo Fisher Scientific, Inc., Waltham, MA, USA). Given the extreme similarity between the *PPO* sequences, specific primers were designed manually by localizing the most different parts after alignment. Primer specificity was checked with Primer Blast and are available in the Supplementary Material. *FaActin* was used as the internal standard for data normalization. Experiments were performed on a StepOne Plus system (Thermo Fisher Scientific, Inc., Waltham, MA, USA).

## 3. Results and Discussion

### 3.1. Selection of PPO Gene Candidates

**Figure 4:**
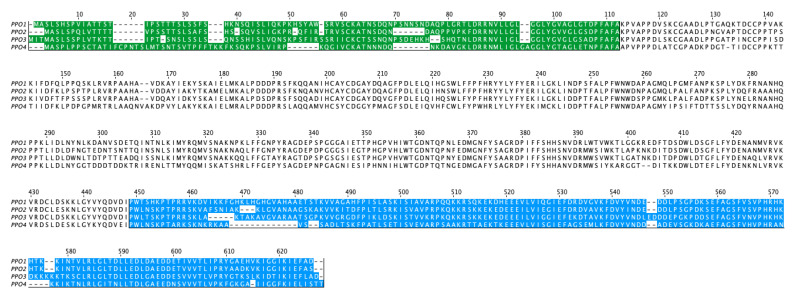
Deduced amino acid sequence alignment of PPO1 (XM_004293480), PPO2 (XM_004293478), PPO3 (XM_004303349), and PPO4 (XM_004293515). Highlighted in green is the transit peptide for integration in the thylakoid membrane, and in blue, the C-terminal domain.

The transcriptomic databases from PacBio next-generation sequencing were used for screening the putative candidates (large green SRX12290367, white SRX12290366, turning SRX12290365, red SRX12290364, and overripe SRX12290363). The sequences for six PPOs were retrieved from NCBI: *PPO1* (XM_004293480), *PPO2* (XM_004293478), *PPO3* (XM_004303349), *PPO4* (XM_004293515), *PPO5* (XM_004295225), and *PPO6* (XM_004291982). The selection was narrowed down to these genes after blasting all of the *PPO* sequences available on NCBI from *Malus x domestica* to a full transcriptomic database from *Fragaria vesca* (Fragaria_vesca_4.0.a2) available on the rosaceae.org website. PrfectBLAST 2.0 was used to scavenge the PacBio databases for similar nucleotide sequences and the program UniPro UGENE 51.0 [40] was used for the visualization of the sequence coverage. Out of the six sequences, only *PPO1*, *PPO2*, *PPO3*, and *PPO4* were present in the fruit. Figure 4 shows the alignment of the deduced amino acid sequence of the four selected *PPOs*. In vivo, the N-terminal sequence, highlighted in green, is cleaved by proteases once the enzyme is successfully translocated to the thylakoid membrane. As it is not part of the active and functional enzyme, it was omitted in the plasmid construct. The deduced amino acid sequences of the PPOs of *Fragaria* × *ananassa* and *Fragaria* × *vesca* were almost identical, as shown exemplarily for PPO2 and PPO4 (Supplementary Figures S1 and S2).

### 3.2. Protein Expression and Purification

The expression of complex proteins such as PPO in *E. coli* is challenging due to their size, presence of disulfide bridges and codon usage diversity. Two initial constructs were designed with *E. coli* codon-optimized sequences to increase the chances of successful expression and yields in the host system. This was effective only for PPO4, while the other three PPOs could not be detected, neither in the soluble nor in the insoluble portion of the lysed cells.

As a first improvement, translocation of the C-terminus 6xHisTag to the N-terminus was tested and proven to be successful for the PPO1 gene, while PPO2 and PPO3 were not expressed. Finally, soluble protein was detected for those two once the codon-optimized sequences were swapped with non-optimized ones and the purification tag was kept at the N-terminus.

**Figure 5:**
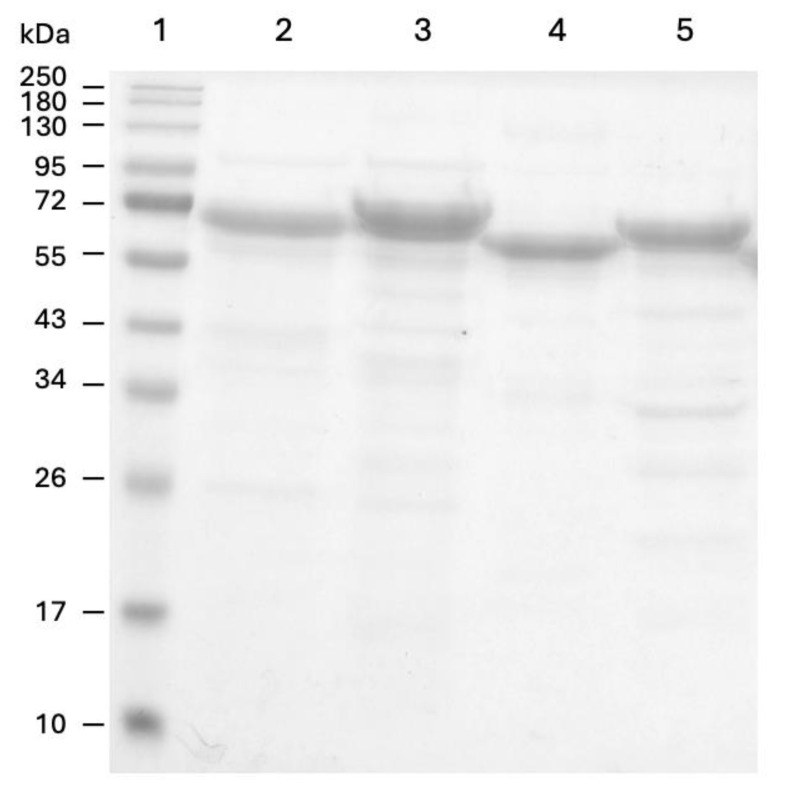
SDS-PAGE gel of purified recombinant PPOs from *E. coli* cultures. 1: Protein size standard, 2: PPO2, 3: PPO3, 4: PPO4, 5: PPO1. Prominent bands are the latent form, while the fainter ones are the result of autoproteolytic activation. In total, 20 µg of enzyme was loaded in each lane.

The enzyme yields were similar for PPO2, PPO3, and PPO4, at 7 mg per liter of culture, whereas PPO1 showed a notable yield of 42 mg per liter. The overall quality of the purified product is shown in Figure 5, and was optimal for enzymatic kinetic characterization, with the major contaminants being fragments derived from the putative autoproteolytic activation of the enzymes.

### 3.3. Enzyme Characterization

#### 3.3.1. Activation of the Latent Enzyme and pH Optimum

**Figure 6:**
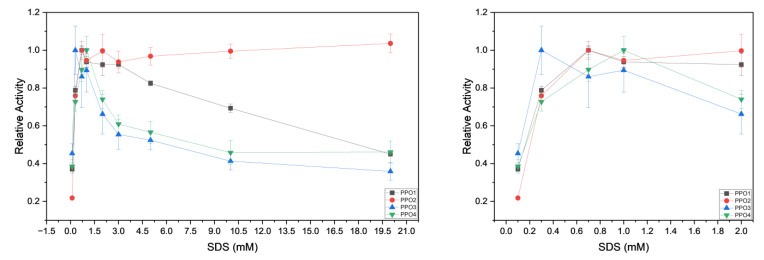
(**Left**) Relative activity for PPO1, PPO2, PPO3, and PPO4 measured at the optimal pH in presence of 10 mM catechol. SDS concentrations ranging from 0.3 mM to 20 mM. (**Right**) Relative activity at concentration from 0.3 mM to 2 mM. At 100% activity, the observed initial apparent velocities were 0.004 for PPO1, 0.003 for PPO2, 0.001 for PPO3, and 0.0004 for PPO4.

Latent PPOs are commonly activated using the detergent SDS [37]. Enzymes were tested in the presence of 10 mM catechol to assess the best SDS concentration for in vitro activation (Figure 6). This is a necessary step because it shortens the time needed for the latent enzyme to become functionally active without removal of the C-terminal domain [41,42]. Detergents, fatty acids, and acidic pH ease the binding energies between the active and shielding domain of the enzyme (Figure 1), allowing permeation of the substrates to the active site [43,44]. In the case of *Fragaria* PPOs, the experimental optimum detergent concentration for PPO1 and PPO2 was 0.7 mM, 1 mM for PPO4, and 0.3 mM for PPO3. The value obtained for PPO3 is probably due to the enzyme instability in solution. The activity values were plotted as the relative activity and were calculated by dividing the apparent velocity at the point of interest with the highest apparent velocity. For example, for PPO1, the highest apparent velocity was at a concentration of 0.7 mM SDS, so at every other concentration, the relative activity was calculated as *V*_x_/*V*_0.7 mM_. PPO2’s maximum apparent velocity was recorded at a detergent concentration of 20 mM, but 0.7 mM was considered more suitable due to excessive foaming when the enzymes and substrate were mixed in the cuvette. The total loss of activity was roughly 3%. The stability of PPO2 at higher detergent concentrations can be explained by point mutations in the sequence that might reinforce electrostatic and hydrophobic interactions between the catalytic and shielding domains, along with a more pronounced occurrence of amino acids that increase a negative electrostatic charge on the outer surface of the enzyme. This would lead to a decrease in binding to the sulfate group of SDS, reducing the overall susceptibility to denaturation.

Apart from detergent activation, pH dependency also plays a big role for the kinetic characterization of PPOs, and is of particular interest as the enzymatic browning of nectars typically occurs in a quite acidic environment. The PPOs were tested at 0.5 pH intervals in the presence of their optimal detergent concentration and 5 mM tyramine as substrate. The PPOs showed quite similar behavior with a pH optimum at 7.0 for PPO1 and PPO2, pH 7.5–9 for PPO4, and pH 8.5 for PPO3. The plotted curves highlight very similar behavior for PPO1 and PPO2 that is probably due to their high sequence similarity. A neutral pH value was chosen as being most suitable for all enzymes during the kinetic assessment, because alkaline solutions increased the auto-oxidation rate of the polyphenol substrates. Enzyme testing at neutral pH improved the overall signal-to-noise ratio in the spectrophotometer, as well as the stability of the enzymes.

**Figure 7:**
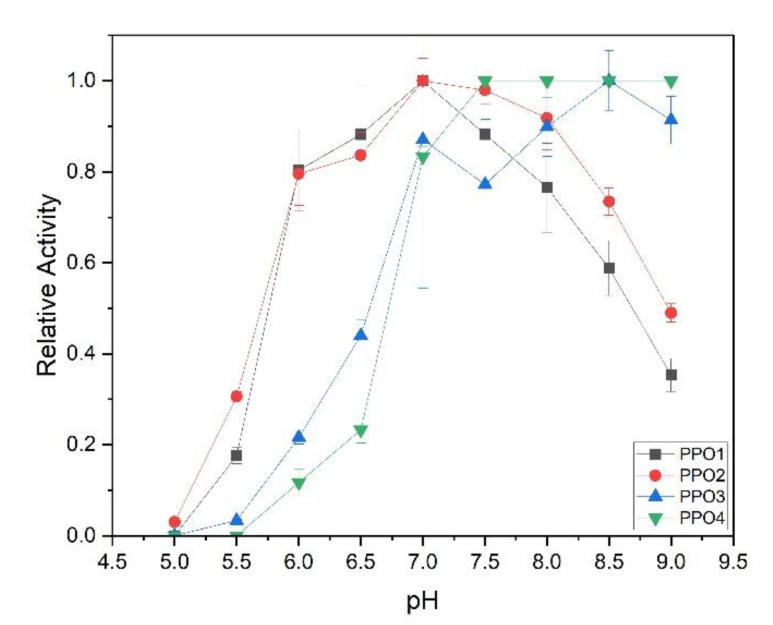
Relative activity of PPOs in the pH ranges from 5.0 to 9.0 with tyramine as a substrate. At 100% activity, the observed initial apparent velocities were 0.0017 for PPO1, 0.0016 for PPO2, 0.001 for PPO3, and 0.0002 for PPO4.

The four SDS-activated recombinant PPOs did not show activity below pH 5.0 (Figure 7). Given that common nectar preparations have a pH around 3.5 [38], which is well below the pH range observed to allow PPO activity, it seems at first sight unlikely that these are responsible for the browning process in nectar. However, the observed low activity at low pHs is probably due to an upward shift of the pH optimum of the enzyme induced by SDS, as shown previously [45,46]. In strawberry fruits, a pH optimum of 4.5 has been observed for PPO activity with much lower, but still relevant, activities at pH 3.5 [35]. Partially purified PPOs from avocado, in comparison, had a pH optimum between 5.5 and 6.5 [47] and aubergine between pH 6.0 and 7.0 [48].

#### 3.3.2. Substrate Specificity of the Recombinant PPOs

The monophenol tyramine was the first substrate tested to assess whether the isoforms possess monophenolase activity. All four PPOs accepted tyramine as a substrate, with comparable substrate affinity to tyramine and the corresponding dihydroxylated dopamine, as reflected by the *K_M_* values (Table 3). But only for PPO4, the catalytic efficiency was higher with tyramine than dopamine. This points to PPO4 being the best catalyst for the conversion of monophenolic substrates, which are rather more present in ripe than unripe strawberry fruits [6,49]. Unfortunately, PPO3 was unstable in solution and the amount used during data collection had to be increased by more than 10 times compared with the other isoforms, and was therefore not used for further kinetic characterization. PPO1, PPO2, and PPO4, in contrast, were quite stable and tested on a set of carefully selected mono- and diphenolic substrates. Tyramine, dopamine, L-DOPA, and catechol were chosen as the most commonly used synthetic substrates for PPO, and catechin and chlorogenic acid, as they are the most abundant diphenols in strawberry fruit. Anthocyanidins and flavonols were accepted as substrates but could not be used for kinetic studies, due to their insufficient aqueous solubility in the required concentrations. Phloretin was also included as it is formed in strawberry and has been shown to have inhibitory effects on PPOs [50]. The calculated turnover values (*k*_cat_), catalytic efficiency (*k*_cat_/*K_M_)*, and affinity (*K_M_*) of each enzyme towards the tested substrates are shown in Table 3.

The catalytic efficiencies of PPO1 and PPO2 were comparable for all the substrates, probably due to the high sequence similarity. Differences are noticeable in both the turnover values and affinity; however, these tend to negate each other, leading to similar efficiencies. PPO4 showed the lowest efficiency towards nearly all substrates, with tyramine as the only outlier. PPO1, PPO2, and PPO4 showed high affinity to the flavan 3-ol catechin as a substrate, and PPO1 and PPO2 had the highest catalytic efficiencies, with values of 1510 s^−1^ mM^−1^ and 772 s^−1^ mM^−1^ for catechin, which are almost doubled catalytic efficiencies, and 5× higher affinities compared to the standard substrate dopamine. Thus, PPO1 and PPO2 of strawberry show a higher specificity for catechin than PPOs from avocado [47]. This could play a role in anthocyanin degradation, as described for strawberry, blueberry, and litchi, potentially through processes like copolymerization [11,51,52]

With chlorogenic acid, which is a quite bulky compound in comparison to the other substrates tested, the catalytic efficiency was significantly lower. This is probably due to the non-planar sugar moiety, which cannot facilitate stabilization of the substrate in the active site by coordination through π-π interactions. The *k*_cat_/*K_M_* values in this case dropped to between 64 s^−1^ mM^−1^ and 153 s^−1^ mM^−1^, which is 2 (PPO2 and PPO4) to 6 (PPO1) times lower than the values obtained with dopamine. Smaller synthetic substrates like catechol also hindered catalysis by lowering the affinity and catalytic efficiency, probably due to the excessive freedom in the active site.

The enzyme activity was also tested with pelargonidin and cyanidin as substrates at pH 3.5 (acidity of strawberry nectars) and 7.0 (experimental pH optimum) to understand if oxidation of the most abundant pigments in the fruit was possible. According to the literature, anthocyanins are not suitable substrates for PPOs, most likely due to the positive charge on the C ring, and this was confirmed for our recombinant enzymes as well [11,53]. In acidic conditions, there was no conversion of the substrate and only a minimal burst in oxygen consumption was recorded during the initial seconds of the reaction at neutral pH. This indicated that the enzymes could oxidize a small amount of the diphenolic pigment cyanidin before being completely deactivated. No conversion of pelargonidin could be detected at both pHs, probably due to the initial long lag phase.

**Figure 8:**
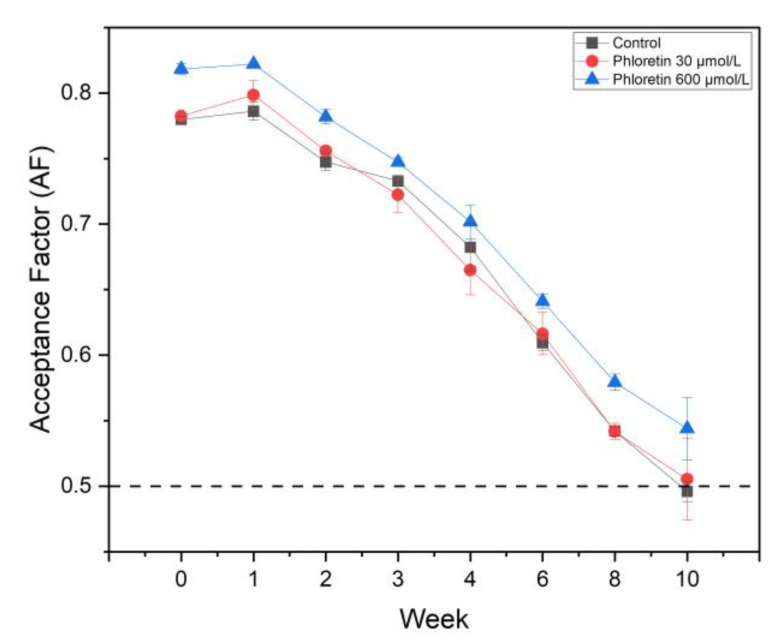
Acceptance Factor [6] determined for nectars containing two concentrations of phloretin, 30 µM (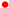) and 600 µM (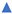), in comparison to a control (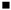). Color stability was measured weekly over a period of 10 weeks. All measurements were performed in technical duplicates and biological triplicates.

In the presence of phloretin, the enzymes demonstrated a high degree of cooperativity, which was positive with PPO1 (*n* = 2.1) and negative with PPO2 and PPO4 (*n* = 0.9 and 0.68, respectively). This behavior has been described previously [54,55] and it was assumed that high concentrations of phloretin induce hysteresis, shortening, or lengthening of the lag phase of catalysis. The strikingly high affinity of the PPOs for phloretin raised the question of whether the addition of phloretin to strawberry nectar could be used as a natural inhibitor of PPOs in juices and nectars. This was tested by adding commercial purified phloretin at two concentrations (30 µmol/L and 60 µmol/L) to freshly prepared strawberry nectar and monitoring color stability by spectrophotometric measurements over 10 weeks (Figure 8). In addition, nectars prepared with commercial clear apple juice, which is a rich source of dihydrochalcones [56,57], and aubergine puree, which is particularly rich in PPO [48,58], respectively, were analyzed (Supplementary Figure S3). The results, illustrated in Figure 8 and Supplementary Figure S3, show the Acceptance Factor (AF), a calculated value that correlates with consumer acceptance of the strawberry nectar color [8]. At a concentration of 600 µmol/L, purified phloretin increased the color stability of strawberry nectar from the beginning, while at a concentration of 30 µmol/L, it had no effect on nectar stability. In the same way, the replacement of water with clear apple juice, which is rich in phloridzin but contains very low concentrations of the free aglycone phloretin, led to a rapid reduction in the AF (Supplementary Figure S3). This decrease, however, could also be attributed to the presence of sugars and other phenolic compounds in the apple juice. The addition of aubergine (Supplementary Figure S3) also resulted in a rapid loss of color stability. This can be explained by the high levels of PPO present in aubergine, which could suggest that PPO can contribute to a loss of color stability if present in high amounts. However, aubergines contain high concentrations of oxidizable compounds, which also could account for the loss of color stability [59]. It is important to note that the achenes were not completely filtered out from the nectar. However, previous studies have reported that the presence or absence of strawberry achenes does not have a significant impact on color stability [12].

### 3.4. RT-qPCR Analysis

**Figure 9:**
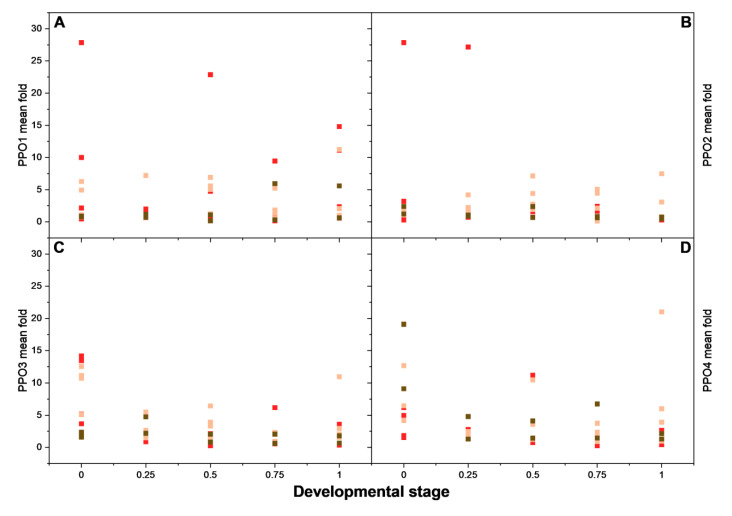
Scatter plot of mean fold change in (A) *PPO1*, (**B**) *PPO2*, (**C**) *PPO3*, (**D**) *PPO4* gene expression with respect to the reference gene Actin (2^−ΔΔCT^). Data of all 10 cultivars/lines are presented, from (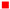) highest stability of lines 190828, 190826, 90886, and 210395, to (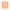) medium stability of cv. Macheraus Marieva, cv Zuckererdbeere Sotschi, cv. Dreistetten, and cv. Diamante, and (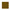) lowest stability of line 90677 and cv. Elsanta.

To investigate whether one of the PPOs is involved in the browning reaction, and the effect of fruit development stages and fruit variety on that reaction, the PPO gene expression was analyzed in five fruit ripening stages of 10 varieties showing differences in color stability (Figure 9, Supplementary Figure S4). This included selected lines resulting from strawberry breeding, as well as traditional and current strawberry cultivars. Lines 190828, 190826, 90886, and 210395 showed the highest stability, cv. Macheraus Marieva, cv. Zuckererdbeere Sotschi, cv. Dreistetten, and cv. Diamante showed moderate color stability, and line 90677 and cv. Elsanta showed a low color stability. Figure 9 shows the distribution of the gene expression of the respective PPOs in comparison to the housekeeping gene *actin* in five developmental stages of the 10 varieties. The expression levels of PPO1 and PPO2 were markedly higher than those of PPO3 and PPO4, confirming their higher expression in strawberry [35].

A correlation with color stability or developmental stage could not be observed for any of the *PPOs* (Figure 9). Although the highest expression values tend to be found in stable lines or cultivars, particularly for *PPOs* 1 and 2, across all developmental stages, other stable varieties also show low expression values. Similarly, despite apparently low expression values amongst varieties with low stabilities, the spread in expression values amongst these varieties would seem to preclude any correlation. Furthermore, nectars prepared from overripe fruits (developmental stage 1) have been shown to be more stable [6,36], whereas none of the *PPOs* display higher expression levels in this stage. Thus, no indication was found that any of the four *PPO* genes expressed in strawberry fruits are particularly involved in the browning reactions. Gene expression data, on the other hand, do not necessarily reflect the overall enzymatic activity, suggesting that the enzymes may have a long half-life once bound to the thylakoid membrane, as supported by the findings of Jia et al. in 2016 [35], which showed stable enzyme activity in fruits, even after a dip in the overall mRNA expression. *PPO* gene expression in relation to the developmental stages is shown in more detail in Supplementary Figure S4, where the relative gene expression was normalized to the values at stage 0. With very few exceptions, the expression of *PPOs* across all 10 cultivars did not exhibit substantial increases at the different ripening stages.

## 4. Conclusions

Our study shows that all four *PPO* genes in strawberries were able to be expressed recombinantly in *E. coli* to a sufficient extent for kinetic characterization. The recombinant enzymes showed their highest activities around neutral pH and activity could be induced by the presence of SDS detergent, even though PPO3 displayed high instability under in vitro conditions. Kinetic data were determined for recombinant PPO1, PPO2, and PPO4. Tyramine could be converted to its quinone, confirming their monophenolase activity. Additionally, the recombinant enzymes showed great affinity and kinetic turnover efficiency towards the flavan 3-ol catechin, and minimal affinity towards cyanidin at neutral pH. The gene expression in 10 different cultivars at five ripening stages did not indicate any significant correlation to color stability, leading to the conclusion that PPO might not be the major player involved in the browning process of strawberry cultivars and juices, even though anthocyanin degradation via reactive quinones might not be ruled out.

## Figures and Tables

**Table 1:** Amounts of *Fa*PPO (µg) used for kinetic characterization and wavelengths used to monitor the catalysis.

*Fa*PPO	Tyramine (470 nm)	Dopamine (465 nm)	L-DOPA (475 nm)	Cathechol (400 nm)	Catechin (440 nm)	Chlorogenic Acid (400 nm)	Phloretin (455 nm)
1	1	0.2	0.1	0.2	0.2	0.5	1
2	1	0.2	0.1	0.2	0.2	0.5	1
4	3	1	1	1	1	2.5	3

**Table 2:** Typical basic physical parameters of strawberry fruits at five ripening stages.

Stage	Code	Height (mm)	Width (mm)	Weight (g)
Green	0	20.3	10.8	2.9
White	0.25	20.6	20.3	7.7
Turning	0.50	20.9	20.9	12.2
Ripe	0.75	30.9	20.9	13.6
Overripe	1.00	30.0	40.0	22.2

**Table 3:** Calculated kinetic parameters for PPO1, PPO2, and PPO4 with the respective standard error. ^1^ Hill coefficient = 2.1; ^2^ Hill coefficient = 0.89; ^3^ Hill coefficient = 0.68. Kinetic parameters not possible to be calculated in reasonable manner.

Substrate	Structure	PPO	*k*_cat_ (s^−1^)	*K_M_* (mM)	*k*_cat_/*K_M_* (s^−1^ mM^−1^)
Tyramine	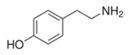	1 2 4	42 ± 1 32.0 ± 0.4 142 ± 20	0.5 ± 0.1 0.6 ± 0.1 1.3 ± 0.1	85 ± 7 54 ± 4 244 ± 45
Dopamine	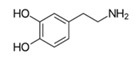	1 2 4	502 ± 18 384 ± 14 125 ± 0.4	0.6 ± 0.1 0.95 ± 0.12 0.99 ± 0.11	827 ± 94 406 ± 39 125 ± 31
L-DOPA	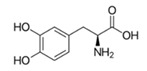	1 2 4	468 ± 8 460 ± 7 30 ± 0.2	2 ± 0.4 5.7 ± 1.9 3.2 ± 1.6	187 ± 47 97 ± 42 17.4 ± 1.5
Catechol	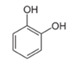	1 2 4	1086 ± 7 827 ± 27 681 ± 10	27 ± 2 7.5 ± 0.6 39 ± 12	292 ± 10 111 ± 7 25 ± 10
Catechin	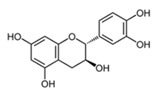	1 2 4	176 ± 11 146 ± 4 142 ± 20	0.12 ± 0.03 0.19 ± 0.04 0.87 ± 0.08	1510 ± 367 772 ± 59 165 ± 11
Chloro-genic acid	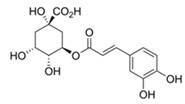	1 2 4	352 ± 20 198 ± 10 82 ± 1	2.7 ± 0.4 1.3 ± 0.2 1.3 ± 0.2	130 ± 67 153 ± 59 64 ± 5
Phloretin	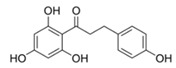	1 2 4	13.5 ± 0.4 ^1^ 35 ± 8 ^2^ - ^3^	0.082 ± 0.004 ^1^ 0.6 ± 0.4 ^2^ - ^3^	165 ± 5 ^1^ 55 ± 18 ^2^ - ^3^

## Data Availability

The original contributions presented in the study are included in the article/Supplementary Material, further inquiries can be directed to the corresponding author.

## Supplementary Materials

The following supporting information can be downloaded at https://www.mdpi.com/article/10.3390/foods14122064/s1, Figure S1: Amino acidic sequence alignment of PPO2 from *Fragaria vesca* (Fv) and *Fragaria* × *ananassa* (Fa); Figure S2: Amino acidic sequence alignment of PPO4 from *Fragaria vesca* (Fv) and *Fragaria* × *ananassa* (Fa); Figure S3: Acceptance Factor measurement for nectars; Figure S4: Gene expression of PPOs; Table S1: RT-qPCR primers used in this study.
