# Supplementary material for: Gene Expression and Enzyme Kinetics of Polyphenol Oxidases in Strawberry and Their Possible Involvement in Enzymatic Browning Reactions in Strawberry Nectar
Source: Foods. 2025 Jun 11;14(12):2064. doi: 10.3390/foods14122064 (PMC12191596; doi:10.3390/foods14122064)
Supplement: Supplementary file 1 [file foods-14-02064-s001.zip › foods-3661978-supplementary.pdf]

foods

Supplementary Materials

1 Supplementary Table

Supplementary Table S1. RT-qPCR primers used in this study

FaPPO1	
For	5′-TGGTCACAAGCTCCACGGCCACG-3′
Rev	5′-CTAGGCAAGTCATCCTCATC-3′
FaPPO2	
For	5′-CTCCCAAAAACAAAGACATCACCG-3′
Rev	5′-CGTTAGCCACACCGAGCTT-3′
FaPPO3	
For	5′-CCCTAGCGATGAACATAAACATTC-3′
Rev	5′-ATCGACTATCTTGTCAGATA-3′
FaPPO4	
For	5′-CCCAGCTGTACTGCTACAATCTTCTG-3′
Rev	5′-TGCCGTATAGACCGCCAGCACCC-3′
FaActin	
For	5′-TCGTGTTGCCCCAGAAGAGC-3′
Rev	5′-CACGATTAGCCTTGGGATTCAG-3′

2 Supplementary Figures

Supplementary Figure S1. Amino acidic sequence alignment of PPO2 from *Fragaria vesca* (Fv) and *Fragaria x ananassa* (Fa)

```
           1                                                   50
Fv_PPO2    MASLSPQLVT TTTVPSSTTS LSAFSHSSQV SLIGKPRQFI RTRVSCKATN
Fa_PPO2    MASLSPQLVT TTTVPSSTTS LSAFSHSSQV SLIGKPRQFI RTRVSCKATN
Consensus  MASLSPQLVT TTTVPSSTTS LSAFSHSSQV SLIGKPRQFI RTRVSCKATN

           51                                                 100
Fv_PPO2    SDQNDAQPPV PKFDRRNVLL GLGGLYGVVG LGSDPFAFAK PVAPPDVSKC
Fa_PPO2    SDQNDAQPPV PKFDRRNVLL GLGGLYGVVG LGSDPFAFAK PVAPPDVSKC
Consensus  SDQNDAQPPV PKFDRRNVLL GLGGLYGVVG LGSDPFAFAK PVAPPDVSKC

           101                                                150
Fv_PPO2    GAADLPNGVA PTDCCPPTPS KIIDFKLPSP TPLRVRPAAH AVDDAYIAKY
Fa_PPO2    GAADLPNGVA PTDCCPPTPS KIIDFKLPSP TPLRVRPAAH AVDDAYIAKY
Consensus  GAADLPNGVA PTDCCPPTPS KIIDFKLPSP TPLRVRPAAH AVDDAYIAKY

           151                                                200
Fv_PPO2    TKAMELMKAL PDDDPRSFKN QANVHCAYCD GAYDQAGFPD LELQIHNSWL
Fa_PPO2    TKAMELMKAL PDDDPRSFKN QANVHCAYCD GAYDQAGFPD LELQIHNSWL
Consensus  TKAMELMKAL PDDDPRSFKN QANVHCAYCD GAYDQAGFPD LELQIHNSWL

           201                                                250
Fv_PPO2    FFPFHRYYLY FYERILGKLI NDPTFALPFW NWDNPAGMQL PALFANPKSP
Fa_PPO2    FFPFHRYYLY FYERILGKLI NDPTFALPFW NWDSPAGMQL PALFANPKSP
Consensus  FFPFHRYYLY FYERILGKLI NDPTFALPFW NWDnPAGMQL PALFANPKSP

           251                                                300
Fv_PPO2    LYDQFRAAAH QPPTLIDLDF NGTEDNTSNT TQINSNLSIM YRQMVSNAKN
Fa_PPO2    LYDQFRAAAH QPPTLIDLDF NGTEDNTSNT TQINSNLSIM YRQMVSNAKN
Consensus  LYDQFRAAAH QPPTLIDLDF NGTEDNTSNT TQINSNLSIM YRQMVSNAKN

           301                                                350
Fv_PPO2    AQLFFGNPYR AGDEPDPGGG SIEGTPHGPV HLWTGDNTQP NFEDMGNFYS
Fa_PPO2    AQLFFGNPYR AGDEPDPGGG SIEGTPHGPV HLWTGDNTQP NFEDMGNFYS
Consensus  AQLFFGNPYR AGDEPDPGGG SIEGTPHGPV HLWTGDNTQP NFEDMGNFYS

           351                                                400
Fv_PPO2    AGRDPIFFSH HSNVDRMWSI WKTLAPKNKD ITDSDWLDSG FLFYDENANM
Fa_PPO2    AGRDPIFFSH HSNVDRMWSI WKTLAPKNKD ITDSDWLDSG FLFYDEDANM
Consensus  AGRDPIFFSH HSNVDRMWSI WKTLAPKNKD ITDSDWLDSG FLFYDE#ANM
```

```
           401                                               450
   Fv_PPO2 VRVKVRDCLE SKNLGYVYQD VDIPWLNSKP TPRRSKVAFS NIAKKLGVAN
   Fa_PPO2 VRVKVRDCLE SKNLGYVYQD VDIPWLNSKP TPRRSKVAFS NIAKKLGVAN
 Consensus VRVKVRDCLE SKNLGYVYQD VDIPWLNSKP TPRRSKVAFS NIAKKLGVAN

           451                                               500
   Fv_PPO2 AAGSKAKVVK ITDFPLTLSR KISVAVPRPK QKKRSKKEKE DEEEILVIEG
   Fa_PPO2 AAGSKPKVVK ITDFPLTLSR KISVAVPRPK QKKRSKKEKE DEEEILVIEG
 Consensus AAGSKaKVVK ITDFPLTLSR KISVAVPRPK QKKRSKKEKE DEEEILVIEG

           501                                               550
   Fv_PPO2 IEFDRDVAVK FDVYINDEDD LPSGPDKSEF AGSFVSVPHR HKHTKKINTV
   Fa_PPO2 IEFDRDVAVK FDVYINDEDD LPSGPDKSEF AGSFVSVPHR HKHTKKINTV
 Consensus IEFDRDVAVK FDVYINDEDD LPSGPDKSEF AGSFVSVPHR HKHTKKINTV

           551                                        594
   Fv_PPO2 LRLGLTDLLE DLDAEDDETV VVTLIPRYAA DKVKIGGIKI EFAS
   Fa_PPO2 LRLGLTDLLE DLDAEDDETV VVTLIPRYAA DKVKIGGIKI EFAS
 Consensus LRLGLTDLLE DLDAEDDETV VVTLIPRYAA DKVKIGGIKI EFAS
```

Supplementary Figure S2. Amino acidic sequence alignment of PPO4 from *Fragaria vesca* (Fv) and *Fragaria x ananassa* (Fa)

PPO4

```
           1                                                  50
   Fv_PPO2 MASPLPPSCT ATIFCPNTSL MTSNTSVTPF FTKKFKSQKP SLVIRPKQGI
   Fa_PPO2 MASPLPPSST ATIFCPNTSL MTSNTSVTPF FTKKFKSQKP SLVIRPKQGI
 Consensus MASPLPPScT ATIFCPNTSL MTSNTSVTPF FTKKFKSQKP SLVIRPKQGI

           51                                                100
   Fv_PPO2 VCKATNNNDQ NKDAVGKLDR RNMLIGLGAG GLYGTAGLET NPFAFAAPVP
   Fa_PPO2 VCKATNNNDQ NKDAVGKLDR RNMLIGLGAG GLYGTAGLET NPFAFAAPVP
 Consensus VCKATNNNDQ NKDAVGKLDR RNMLIGLGAG GLYGTAGLET NPFAFAAPVP

           101                                               150
   Fv_PPO2 PPDLATCGPA DKPDGTTIDC CPPKTTTIID FKLPDPGPMR TRLAAQNVAK
   Fa_PPO2 PPDLATCGPA DKPDGTTIDC CPPKTTTIID FKLPDPGPMR TRLAAQNVAK
 Consensus PPDLATCGPA DKPDGTTIDC CPPKTTTIID FKLPDPGPMR TRLAAQNVAK

           151                                               200
   Fv_PPO2 DPVYLAKYKK AIELMRALPD DDPRSLAQQA MVHCSYCDGG YPMAGFSDLE
   Fa_PPO2 DPVYLAKYKK AIELMRALPD DDPRSLAQQA MVHCSYCDGG YPMAGFSDLE
 Consensus DPVYLAKYKK AIELMRALPD DDPRSLAQQA MVHCSYCDGG YPMAGFSDLE

           201                                               250
   Fv_PPO2 IQVHFCWLFY PWHRLYLYFY EKIMCKLIDD PTFALPFWNW DAPAGMYIPS
   Fa_PPO2 IQVHFCWLFY PWHRLYLYFY EKIMCKLIDD PTFALPFWNW DAPAGMYIPS
 Consensus IQVHFCWLFY PWHRLYLYFY EKIMCKLIDD PTFALPFWNW DAPAGMYIPS

           251                                               300
   Fv_PPO2 IFTDTTSSLY DQYRNAAHQP PKLLDLNYGG TDDDTDDKTR IRENLTTMYQ
   Fa_PPO2 IFTDTTSSLY DQYRNAAHQP PKLLDLNYGG TDDDTDDKTR IRENLTTMYQ
 Consensus IFTDTTSSLY DQYRNAAHQP PKLLDLNYGG TDDDTDDKTR IRENLTTMYQ

           301                                               350
   Fv_PPO2 QMISKATSHR LFFGEPYSAG DEPNPGAGNI ESIPHNNIHL WTGDPTQTNG
   Fa_PPO2 QMISKATSHR LFFGEPYSAG DEPNPGAGNI ESIPHNNIHL WTGDPTQTNG
 Consensus QMISKATSHR LFFGEPYSAG DEPNPGAGNI ESIPHNNIHL WTGDPTQTNG

           351                                               400
   Fv_PPO2 EDMGAFYSAG RDPIFYSHHA NVDRMWSIYK ARGGTDITKK DWLDTEFLFY
   Fa_PPO2 EDMGAFYSAG RDPIFYSHHA NVDRMWSIYK ARGGTDITKK DWLDTEFLFY
 Consensus EDMGAFYSAG RDPIFYSHHA NVDRMWSIYK ARGGTDITKK DWLDTEFLFY
```

```
           401                                                  450
  Fv_PPO2  DENKNLVRVK VRDSLDESKL GYKYQDVEIP WLNSKPTARK SKNKRKAAVS
  Fa_PPO2  DENKNLVRVK VRDSLDESKL GYKYQDVEIP WLNSKPTARK SKNKRKAAVS
Consensus  DENKNLVRVK VRDSLDESKL GYKYQDVEIP WLNSKPTARK SKNKRKAAVS

           451                                                  500
  Fv_PPO2  SADLTSKFPA TLSETISVEV ARPSAAKRTT AEKTKEEEVL VISGIEFAGS
  Fa_PPO2  SADLTSKFPA TLSETISVEV ARPSAAKRTT AEKTKEEEVL VISGIEFAGS
Consensus  SADLTSKFPA TLSETISVEV ARPSAAKRTT AEKTKEEEVL VISGIEFAGS

           501                                                  550
  Fv_PPO2  EMLKFDVYVN DDADEVSGKD KAEFAGSFVH VPHRANKKIK TNLRLGITNL
  Fa_PPO2  EMLKFDVYVN DDADEVSGKD KAEFAGSFVH VPHRANKKIK TNLRLGITNL
Consensus  EMLKFDVYVN DDADEVSGKD KAEFAGSFVH VPHRANKKIK TNLRLGITNL

           551                                       587
  Fv_PPO2  LTDLGAEEDN SVVVTLVPKF GKGAIIGGFK IELISTT
  Fa_PPO2  LTDLGAEEDN SVVVTLVPRA NSKLT..... .......
Consensus  LTDLGAEEDN SVVVTLVPra nkgai..... .......
```

Supplementary Figure S3. Acceptance Factor measurement for nectars. (●) Prepared with additions of aubergine 1:5 w:v, (▲) in which water was replaced with apple juice compared to a (■) control. Data were measured weekly for 10 weeks, all measurement were done in triplicate.

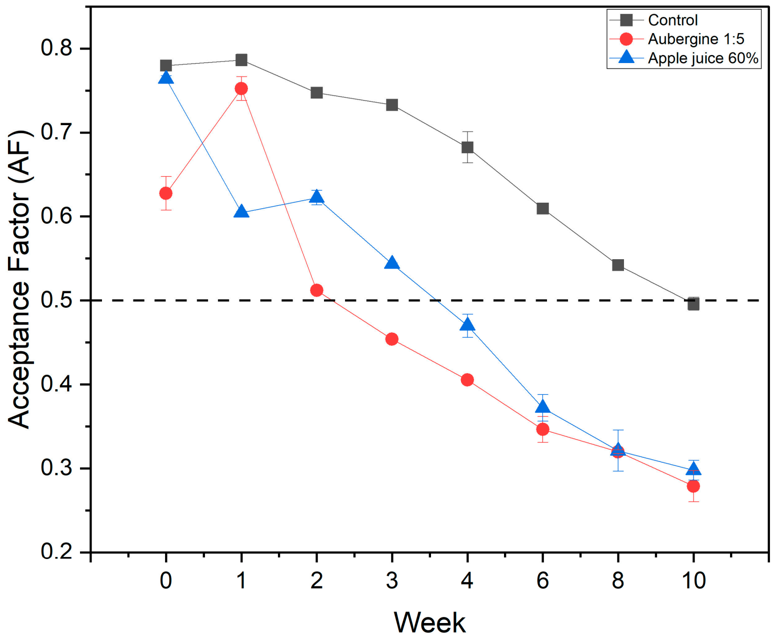

Supplementary Figure S4. Gene expression of *PPOs*. Gene expression of all 10 cultivars, expressed as Mean Fold change of *PPO1, PPO2, PPO3, PPO4* normalized to stage 0. Data of all 10 cultivars are presented in a separated plot based on relevant stability with most stable (A) line 190828 to the least stable (J) cv. Elsanta based on enzyme and ripening stage.

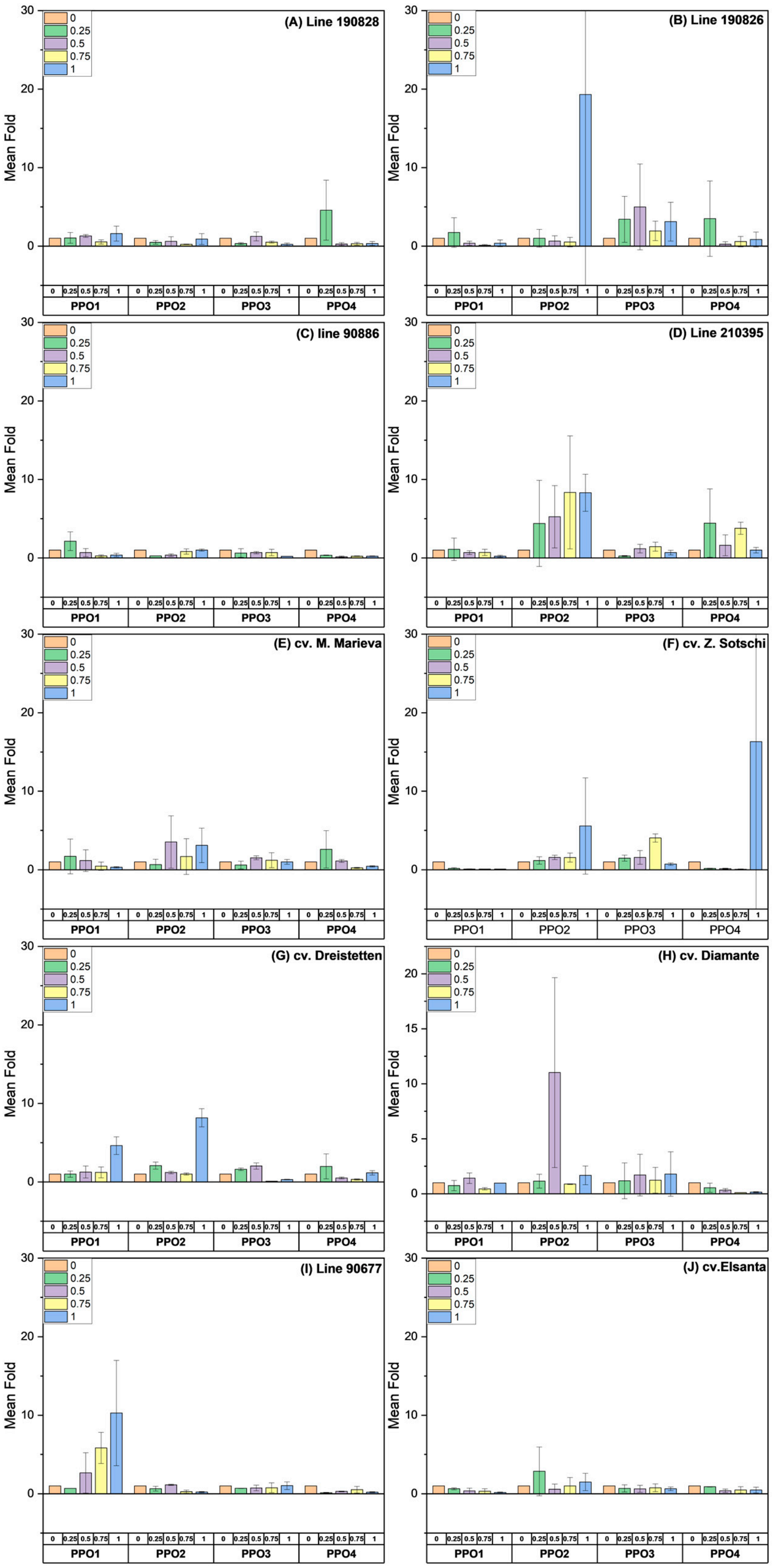

Disclaimer/Publisher's Note: The statements, opinions and data contained in all publications are solely those of the individual author(s) and contributor(s) and not of MDPI and/or the editor(s). MDPI and/or the editor(s) disclaim responsibility for any injury to people or property resulting from any ideas, methods, instructions or products referred to in the content.
